# A deep learning model for brain age prediction using minimally preprocessed T1w images as input

**DOI:** 10.3389/fnagi.2023.1303036

**Published:** 2024-01-08

**Authors:** Caroline Dartora, Anna Marseglia, Gustav Mårtensson, Gull Rukh, Junhua Dang, J-Sebastian Muehlboeck, Lars-Olof Wahlund, Rodrigo Moreno, José Barroso, Daniel Ferreira, Helgi B. Schiöth, Eric Westman, for the Alzheimer’s Disease Neuroimaging Initiative, the Australian Imaging Biomarkers and Lifestyle Flagship Study of Ageing, the Japanese Alzheimer’s Disease Neuroimaging Initiative, the AddNeuroMed Consortium

**Affiliations:** ^1^Division of Clinical Geriatrics, Center for Alzheimer Research, Department of Neurobiology, Care Sciences and Society, Karolinska Institutet, Stockholm, Sweden; ^2^Department of Surgical Sciences, Functional Pharmacology and Neuroscience, Uppsala University, Uppsala, Sweden; ^3^Department of Biomedical Engineering and Health Systems, KTH Royal Institute of Technology, Stockholm, Sweden; ^4^Facultad de Ciencias de la Salud, Universidad Fernando Pessoa Canarias, Las Palmas, España; ^5^Department of Neuroimaging, Centre for Neuroimaging Sciences, Institute of Psychiatry, Psychology and Neuroscience, King’s College London, London, United Kingdom

**Keywords:** brain age, neurodegeneration, normal ageing, CNN, UK Biobank, ageing prediction

## Abstract

**Introduction:**

In the last few years, several models trying to calculate the biological brain age have been proposed based on structural magnetic resonance imaging scans (T1-weighted MRIs, T1w) using multivariate methods and machine learning. We developed and validated a convolutional neural network (CNN)-based biological brain age prediction model that uses one T1w MRI preprocessing step when applying the model to external datasets to simplify implementation and increase accessibility in research settings. Our model only requires rigid image registration to the MNI space, which is an advantage compared to previous methods that require more preprocessing steps, such as feature extraction.

**Methods:**

We used a multicohort dataset of cognitively healthy individuals (age range = 32.0–95.7 years) comprising 17,296 MRIs for training and evaluation. We compared our model using hold-out (CNN1) and cross-validation (CNN2–4) approaches. To verify generalisability, we used two external datasets with different populations and MRI scan characteristics to evaluate the model. To demonstrate its usability, we included the external dataset’s images in the cross-validation training (CNN3). To ensure that our model used only the brain signal on the image, we also predicted brain age using skull-stripped images (CNN4).

**Results:**

The trained models achieved a mean absolute error of 2.99, 2.67, 2.67, and 3.08 years for CNN1–4, respectively. The model’s performance in the external dataset was in the typical range of mean absolute error (MAE) found in the literature for testing sets. Adding the external dataset to the training set (CNN3), overall, MAE is unaffected, but individual cohort MAE improves (5.63–2.25 years). Salience maps of predictions reveal that periventricular, temporal, and insular regions are the most important for age prediction.

**Discussion:**

We provide indicators for using biological (predicted) brain age as a metric for age correction in neuroimaging studies as an alternative to the traditional chronological age. In conclusion, using different approaches, our CNN-based model showed good performance using one T1w brain MRI preprocessing step. The proposed CNN model is made publicly available for the research community to be easily implemented and used to study ageing and age-related disorders.

## 1 Introduction

In recent years, the concept of an individual’s biological age—which can differ from the person’s chronological age—has sparked great interest in the medical research community, as ageing is a significant risk factor for several age-related health conditions and mortality. There is also substantial heterogeneity in health outcomes amongst individuals of the same chronological age (Jylhävä et al., 2017). During the past decades, the research highlighted that the biological ageing process varies between people because of the complex interplay between genetic and environmental factors, such as lifestyle behaviours (Cole et al., 2017, 2019; Fratiglioni et al., 2020). Given the ongoing changes in the body and brain throughout the ageing process, chronological age stands out as a key risk factor for mortality, chronic diseases, and functional impairment (Jylhävä et al., 2017). Various age-related changes in the brain are closely linked to the development of several neurodegenerative disorders, including Alzheimer’s disease (AD) and vascular dementia (Hou et al., 2019). Like other age-related health conditions and also in the dementia field, there is significant heterogeneity in the manifestation of the symptoms as well as underlying brain pathology between people of the same chronological age (Ferreira et al., 2020). Therefore, quantifying the biological age could be a useful additional metric than the traditional chronological age to identify individuals at risk of developing age-related diseases (Cole et al., 2019; Tian et al., 2023).

Parallel advancements in neuroscience and computational science have enabled researchers to develop novel algorithms to determine the biological age of the brain. A biological marker of brain age will enable us to adjust neuroimaging studies for the person’s biological age instead of chronological age, capturing anatomical and functional heterogeneities present in groups of healthy individuals. Another advantage is that this could lead to a deeper understanding of pathological ageing mechanisms, which can culminate in dementia. Dementia is a multifactorial syndrome in which decades of accumulating neuropathology precedes clinical manifestation (Jack et al., 2010). The loss of neurons and synapses during the preclinical and prodromal stages can lead to brain atrophy and, therefore, to “older-looking” brains (when biological brain age, i.e., predicted age, is higher than chronological age) (Cole and Franke, 2017; Bashyam et al., 2020; Cole, 2020; Elliott et al., 2021). In contrast, some individuals will show a higher chronological age than the biological brain age, thus showing a “younger-looking” brain, which could reflect relatively preserved brain structures (e.g., brain maintenance and/or cognitive reserve) (Cole et al., 2019; Stern et al., 2020). With the unprecedented growth of the elderly population worldwide and the expected increase in dementia cases (WHO guidelines, 2019), a biological marker of brain age could play a key role in dementia prevention (Brusini et al., 2022).

In recent years, several brain age models have been developed using different methods (Bocancea et al., 2021; Baecker et al., 2021a). Previous studies employed machine (Franke et al., 2010; Franke and Gaser, 2012; Cole et al., 2017; Hwang et al., 2021) and deep learning techniques, with a focus on convolutional neural networks (CNN) (Cole et al., 2017; Jonsson et al., 2019; Kolbeinsson et al., 2019; Liang et al., 2019; Bintsi et al., 2020; Lam et al., 2020; Niu et al., 2020; Gupta et al., 2021; Wood et al., 2022). These approaches achieved good performance regarding mean absolute error (MAE) between 2 and 6 years, with CNNs exhibiting superior results using images with few image preprocessing steps. However, the model type and the input choice varied across these studies that used preprocessed magnetic resonance imaging (MRI) data (T1-weighted, T1w, or T2w), going through normalisation, corrections, segmentation steps, or image feature extraction. Such a chain of steps is challenging to implement in research and, in the long term, in clinical settings due to time- and resource-consuming constraints.

Developing a model to predict “biological brain age” hinges on selecting training data. Typically, a model is trained on neuroimaging data of healthy individuals sourced from one or multiple cohorts, encompassing a broad age range. The “ideal” dataset would include: (1) detailed information and clinical data of study participants in order to be as comprehensive as possible with the selection criteria; (2) a large set of images, which are required to train a CNN model (Sajedi and Pardakhti, 2019); (3) participants with a diverse demographical background and a large, preferably uniform, age distribution to apply the developed model in more datasets (i.e., increase generalisability); (4) images acquired with a wide range of MRI scanners and protocols to improve generalisation to new unseen data of the model (Mårtensson et al., 2020), and (5) longitudinal data to ensure that the model does not predict a lower age at a later time point. Although initiatives to gather large-scale population-based datasets are ongoing (e.g., UK Biobank), to the best of our knowledge, no existing cohort possesses all the five characteristics listed above.

In this study, we aimed to develop and validate a CNN model based on brain images that uses one preprocessing step (i.e., rigid registration of T1w MRIs to the Montreal Neurological Institute—MNI—template space) for brain age prediction when using external datasets. This minimal preprocessing feature has the advantage and strength of simplifying the model’s implementation and increasing accessibility in research settings. When publicly available, the model can be quickly used for any T1w MRI scan without time- and resource-consuming preprocessing steps. To evaluate our model, we used a large dataset of cognitively healthy individuals from six cohorts to address the “ideal” dataset criteria. The CNN model was compared using hold-out and cross-validation approaches. To verify the model’s generalisability, we tested the abovementioned approaches using two external datasets containing different scanners and demographic characteristics from the training set. Furthermore, we included the two cohorts used as external datasets in the cross-validation loop to verify the model’s usability with different cohorts. Finally, we employed the cross-validated model to predict brain age in skull-stripped images to ensure our model accurately predicted based on the brain image signal. We then evaluated the model’s performance using two external datasets.

## 2 Materials and methods

### 2.1 Study population

**Figure 1:**
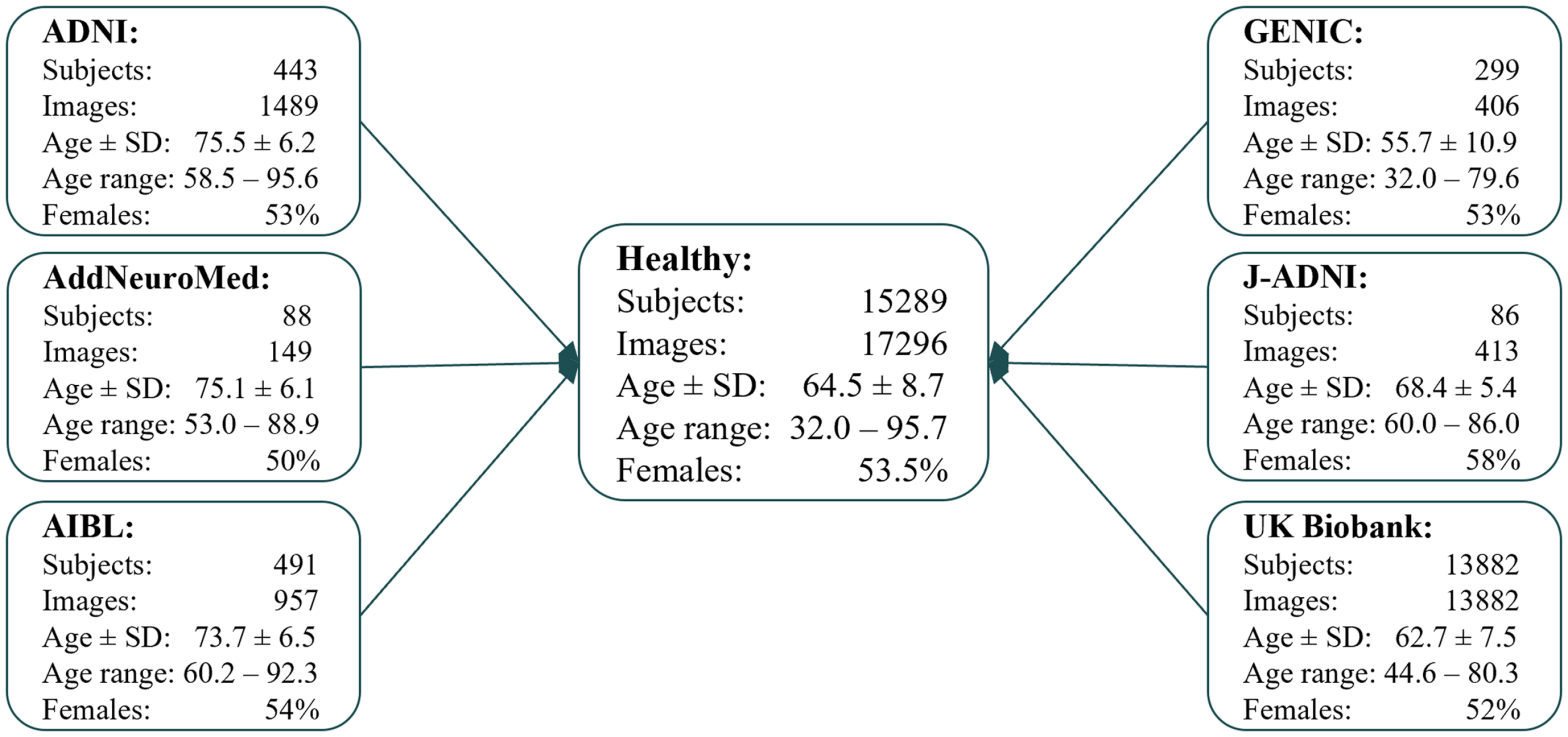
Flowchart describing the data included in this study, which were available in our database system at the time of the study, for the different cohorts. Where age ± SD is the mean age and standard deviation. Age ± SD and age range are in years.

For this study, we included 17,296 T1w MRIs from 15,289 (1,176 are 1.5 T and 16,120 are 3 T) cognitively healthy participants from six cohorts: the Alzheimer’s Disease Neuroimaging Initiative (ADNI), the Australian Imaging, Biomarker & Lifestyle Flagship Study of Ageing (AIBL), the AddNeuroMed, the Group of Neuropsychological Studies from the Canary Islands (GENIC, from Grupo de Estudios Neuropsicologicos de las Islas Canarias), the Japanese ADNI, and the UK Biobank (Figure 1). The description of each cohort and information about image acquisition protocols and scanners are in Supplementary material, Section 1. A cognitively healthy status was defined based on the absence of dementia, mild cognitive impairment, and other neurological and psychiatric disorders. Furthermore, individuals had to have a clinical dementia rating (CDR) score equal to zero, a mini-mental state examination (MMSE) score ≥ 24, self-reported good health (this last when available), or ICD-9 or − 10 (details on the used ICD codes can be found in Supplementary Table S1), depending on the available data in each cohort.

### 2.2 Convolutional neural networks

The implemented supervised CNN uses PyTorch’s deep learning framework (Paszke et al., 2019). The network architecture of our model was based on the ResNet architecture (He et al., 2016), with 26 layers in total but with 3D kernels. Each convolutional operation is followed by batch normalisation and a Rectified Linear Unit (ReLU) activation. The network was trained for 20 epochs with stochastic gradient descent and an initial learning rate of λ = 0.002 that decreases by a factor of 10 every five epochs. We used five independent models during CNN development and the trained networks as an ensemble model. Data augmentation was performed during model training in 70% of the training set and included random scaling, cropping offsets, rotations (−5 to 5 degrees), affine, and gamma transformations (ranging from 0.5 to 2). In the data augmentation process, each image was cropped to a dimension of 80 × 96 × 80 voxels with 2 × 2 × 2 mm^3^ resolution, thresholded to the 5th and 95th percentiles of the voxel values, and scaled so that all voxels’ values were in the interval [−1, 1].

**Figure 2:**
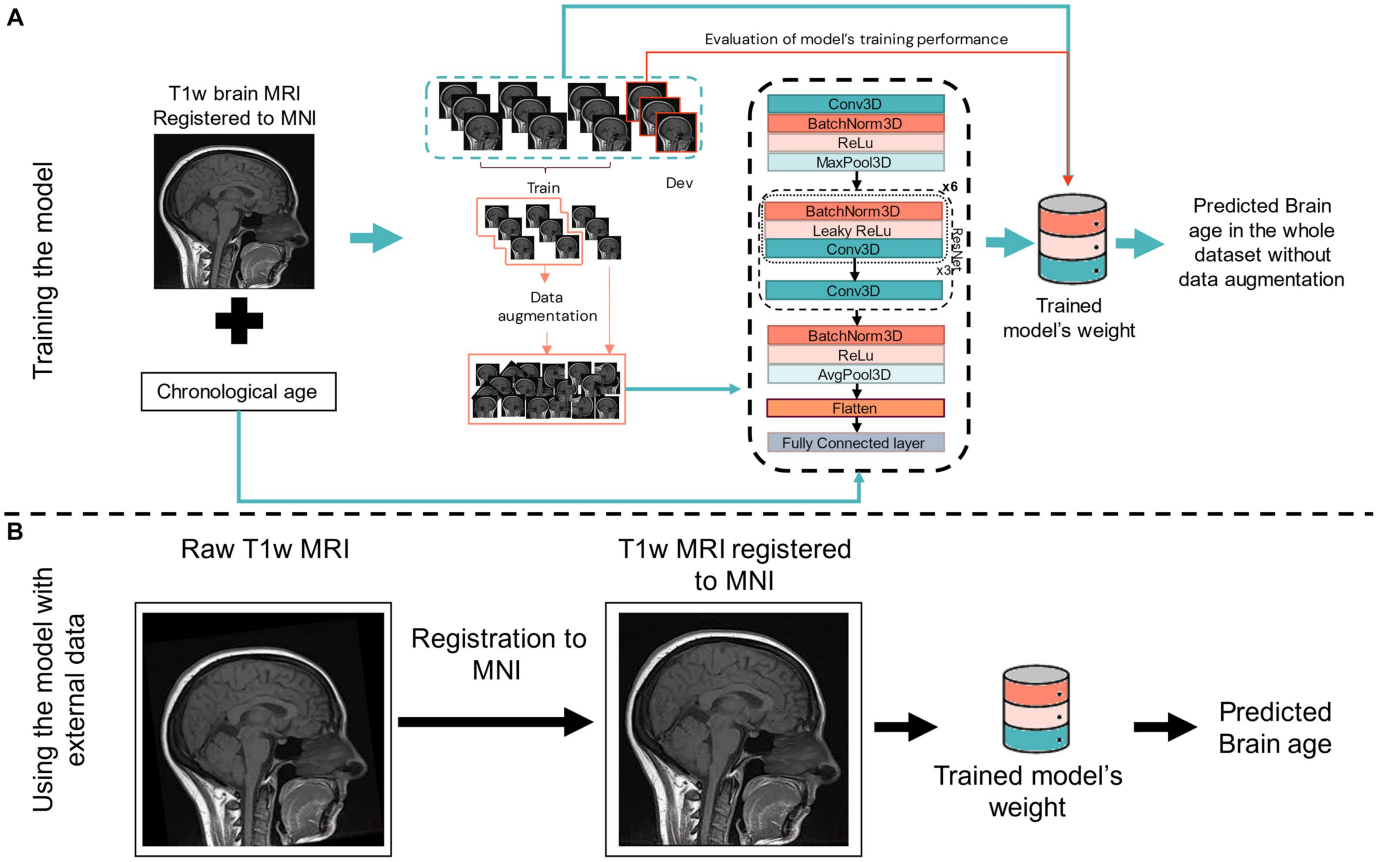
Scheme of our 3D CNN for training **(A)** and using for external dataset **(B)**. In **(A)**, the input is a T1w brain MRI previously registered to the MNI space, the subject’s chronological age, and the output is the predicted brain age of the subject. Training and developing (dev) datasets are also shown, with data augmentation being performed in 70% of the training set. In **(B)**, the input is only a T1w MRI previously registered to the MNI space before using the trained network (model’s weight) to predict brain age in the new and unseen data of an external dataset. Conv3D, 3D convolution; BatchNorm3d, 3D batch normalisation; Dev, development dataset (for testing model’s performance while training); (Leaky)ReLu, (leaky) rectified linear unit; MaxPool3D, 3D max pooling; AvgPool3D, 3D average pooling; FC, fully connected layer; ResNet, residual network block.

For training the model, a T1w brain MRI registered to the MNI space and the individual’s chronological age were used. We streamlined the image preprocessing to improve the model’s accessibility and processing speed. The sole preprocessing step involved is a rigid registration (with six degrees of freedom) of the T1w MPRAGE MRI to the MNI template space using FSL FLIRT 6.0 (FMRIB’s Linear Image Registration Tool) for training the model and testing in external datasets. A rigid registration is more straightforward and quicker than an elastic registration. Omitting this step resulted in worse performance despite implementing heavy data augmentation in the training process (data not shown). Figure 2 shows a schematic representation of the CNN model, its input for training, and when using it with external datasets.

**Figure 3:**
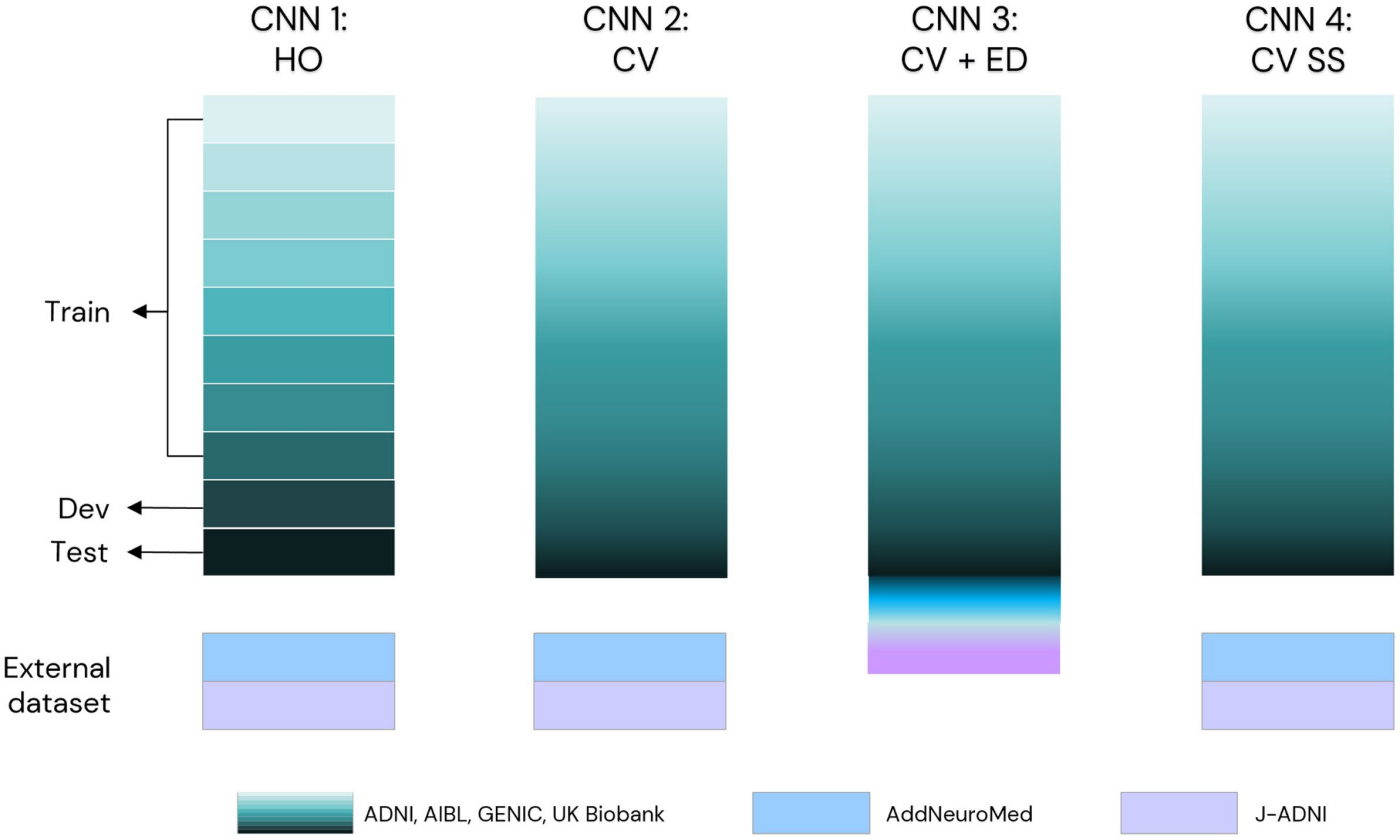
Scheme of evaluation of our CNN model in this study. We primarily used ADNI, AIBL, GENIC, and the UK Biobank to develop our model (turquoise scale). CNN1 works in a hold-out approach (data split: 80% train, 10% development, 10% test set, each set of the data indicated by arrows). CNN2 was trained as a 10-fold cross-validation model using the data of the four primary cohorts (turquoise scale) in the training loop. To evaluate the performance of these two models, we used AddNeuroMed (light blue) and J-ADNI (lilac) as external test sets. In CNN3, we added the external test sets in the 10-fold cross-validation. For comparison reasons, we evaluated our CNN2 scheme with skull-stripped T1w MRIs (CNN4). HO, hold-out; CV, cross-validation; ED, external test set; SS, skull-stripped images.

Four separate models were developed in this project: one model based on a hold-out approach of CNN (CNN1) and three models based on a cross-validation approach (CNN2–4). Figure 3 displays the evaluation process scheme of our CNN model. Each one of the turquoise rectangles represents 1/10 of the primary dataset, composed of 16,734 raw MRIs from ADNI, AIBL, GENIC, and UKB cohorts. Light blue rectangles indicate the 149 MRIs from AddNeuroMed cohort, whereas lilac rectangles indicate the 413 MRIs from J-ADNI. The CNN1 model is based on a hold-out approach with the training (80%), development (10%), and test (10%) datasets indicated by the arrows. CNN2 and CNN4 models incorporated all data from ADNI, AIBL, GENIC, and UKB cohorts in their cross-validation loop, while AddNeuromed and J-ADNI were used for external validation. The CNN3 model was similar to CNN2 and 4, except that AddNeuroMed and J-ADNI were also incorporated in the cross-validation loop—thus, no external datasets were used for model evaluation.

#### 2.2.1 Hold-out approach

Our CNN-based model was first trained in a hold-out fashion (CNN1). To not violate the test set and ensure comparability between the models, the primary dataset, composed of 16,734 MRIs from ADNI, AIBL, GENIC, and UKB cohorts, was randomly split into a training (N*_img_* = 15,052, N*_subj_* = 13,612, subsequently split into internal validation set for the development of each model) and a hold-out test (N*_img_* = 1,682, N*_subj_* = 1,503) set. If subjects had undergone multiple scans, all their images were assigned to the same set. The test set was evaluated after a satisfactory performance on the internal validation set to reduce the risk of model overfitting. The data distribution in train, development, and test sets for each cohort can be found in Supplementary Figure S1. After training the CNN1 model, we applied this model in the AddNeuroMed and J-ADNI cohorts to assess the model’s performance and generalisability in external datasets.

#### 2.2.2 Cross-validated approach

To allow comparability to the hold-out model (CNN1), we used the same 16,734 MRIs from ADNI, AIBL, GENIC, and UK Biobank in the cross-validation approach training loop (CNN2). Stratification by cohort was applied in splitting the 10-fold for training and testing. After 10-fold cross-validation, the trained model was evaluated in AddNeuroMed and J-ADNI (external cohorts). Furthermore, to ensure that our model’s prediction was based on the brain and not on other features (e.g., head shape), we trained a 10-fold cross-validation model using skull-stripped brain images (CNN4). For CNN4 model image input, Freesurfer 6.0.0 was used to perform skull-stripping, applying the algorithm recon-all, and select the image generated before brain parcellation (brain.finalsurfs.mgz). Images were motion- and bias-corrected, transformed to Tailarach space, intensity-normalised, and skull-stripped. To reduce the size of the final processed image and for comparative reasons, all skull-stripped images were rigidly registered to the MNI space. Similar to CNN2, the skull-stripped CNN4 model was externally validated in AddNeuroMed and J-ADNI. Finally, to ensure the cross-validated model’s usability when including more diverse data, we trained a last model, CNN3, that included all cohorts (ADNI, AIBL, GENIC, and UK Biobank plus AddNeuroMed and J-ADNI) to the ensemble of images within the 10-fold cross-validation.

### 2.3 Analyses

#### 2.3.1 Model performance

Model performance was assessed using the MAE, defined as:


$$\text{MAE}=\frac{1}{N}\sum_{i}^{N}\left|{\hat{y}}_{i}-y_{c,i}\right|$$

where $y_{c,i}$ is the chronological age of participant i and ${\hat{y}}_{i}$ the predicted age. MAE’s values close to zero indicate the model’s good performance, with predicted brain age being similar or almost equal to chronological age. Consequently, the evaluation of the model’s performance includes assessing the distribution and correlations between chronological and predicted brain age, in addition to MAE. Adjusting for age-dependent predicted brain age differences from the chronological age (brain age difference/gap—BAG) is problematic and can artificially inflate model performance (Butler et al., 2021). This can be illustrated with a model that, regardless of input data, only outputs a single predicted age of, e.g., 70. This will yield a suboptimal MAE, but when “correcting” for age, the MAE will be 0 between predicted and chronological age. Given the widespread use of the UK Biobank in the literature as a common dataset for training and evaluating brain age models, we conducted additional assessments of our model’s performance using only the UK Biobank cohort.

#### 2.3.2 Relevant regions for brain age prediction

To explain the model’s brain age prediction, we generated 3D gradient-based saliency maps of each subject using the SmoothGrad (Smilkov et al., 2017) algorithm. Salience maps visualise the important voxels in individual predictions based on the computation of the gradient of the prediction with respect to the smoothed image. For gradient computation, we used the image with 15% noise added. The 3D gradient maps were averaged through the whole image sample, and only 1% of the higher salient values were shown to verify the most critical regions (Levakov et al., 2020; Mouches et al., 2022). For individual extrapolation, we plotted the 1% normalised higher values of the salience maps and overlayed them onto an arbitrary T1w brain MRI. The salience maps are presented according to their brain age difference in the CNN1 model, calculated from the chronological age, from −8 to +8 years of difference from the chronological age.

#### 2.3.3 Differences in cortical thickness across age groups based on chronological and predicted brain age

To analyse the influence of correcting an individual’s brain age in neuroimaging studies, we visualise how brain age predictions are related to cortical thickness values. We ran surface group analysis with QDEC (Query, Design, Estimate, Contrast) in FreeSurfer 6.0.0. We used a smoothing kernel of full width at half maximum of 10 mm, used sex as a covariate, and adjusted for false discovery rate at a threshold of 0.05. We grouped subjects of ages 60, 65, 70, 75, and 80 (±1 year) based on chronological and predicted ages. These groups were contrasted to a reference group of 55 ± 1 years old (based on corresponding chronological or predicted brain age) individuals in the general linear model. Since these groups are of different sizes—and *p-values* are influenced by group size—we present figures overlayed with *z*-scores.

## 3 Results

### 3.1 Model performance

**Figure 4:**
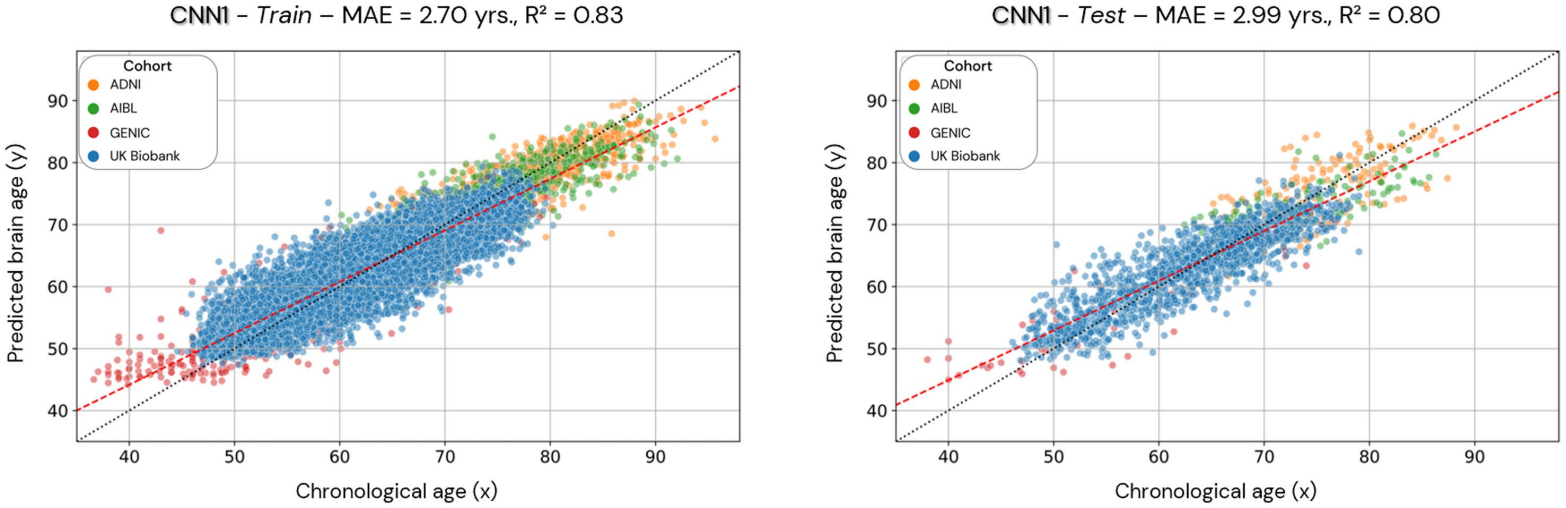
Scatterplots of the predicted brain age in the CNN1 model (hold-out approach) in the training and test datasets. Each colored dot represents an individual, and each color is a different cohort. The red line is a linear regression based on the predicted brain age.

**Figure 5:**
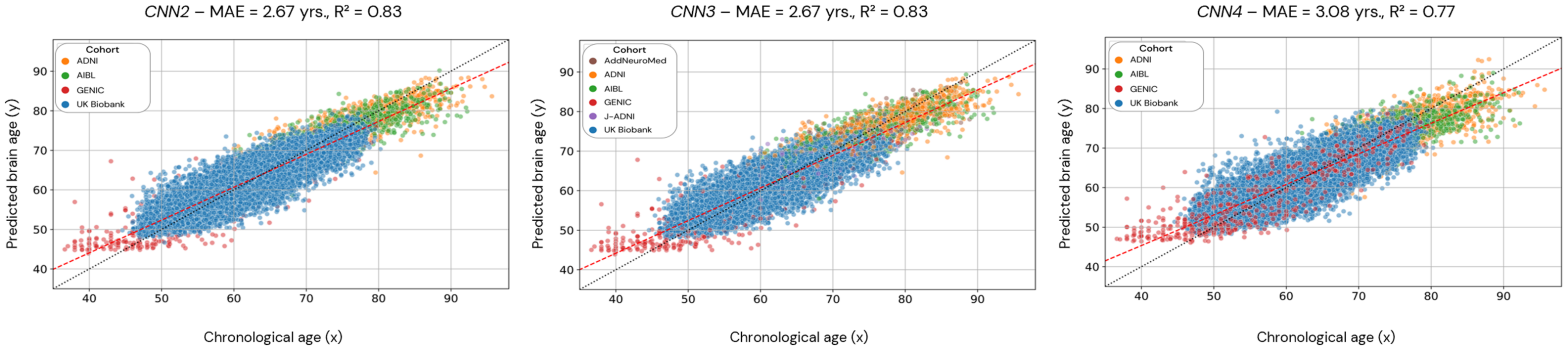
Scatterplots of the predicted brain age in CNN2, 3, and 4 (cross-validation approach). Each dot represents an individual, and the color code used for each cohort is presented in the legend. CNN2 was run using 4 cohorts, CNN3 with 6 cohorts, and CNN4 with 4 cohorts but with skull-stripped images. The red line is the linear regression based on the predicted brain age.

**Figure 6:**
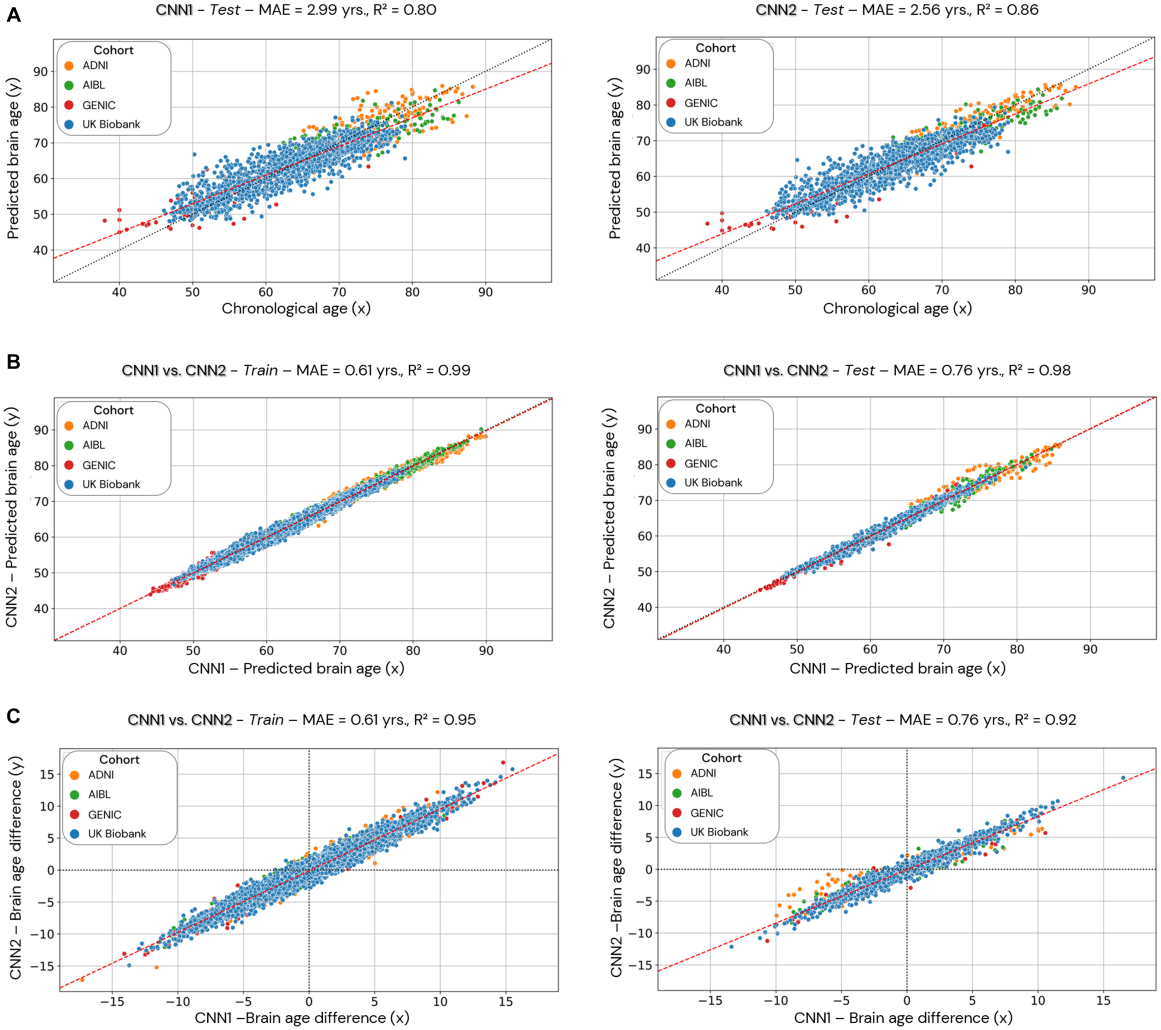
Comparison of the CNN1 (hold-out approach) and CNN2 (cross-validation approach) models. Data used for the comparison is based only on the individuals used in the test dataset of CNN1. In **(A)**, we present the test dataset’s predicted brain age (*y*-axis) for both models correlated to chronological age (*x*-axis). In **(B)**, we present the correlation between the predicted brain age estimated by the two models (CNN1 on the *x*-axis and CNN2 on the *y*-axis) in the training (left) and test (right) datasets split by CNN1. In **(C)**, we present the correlation between the brain age difference estimated by the two models (CNN1 on the *x*-axis and CNN2 on the *y*-axis) in the training and test datasets.

**Figure 7:**
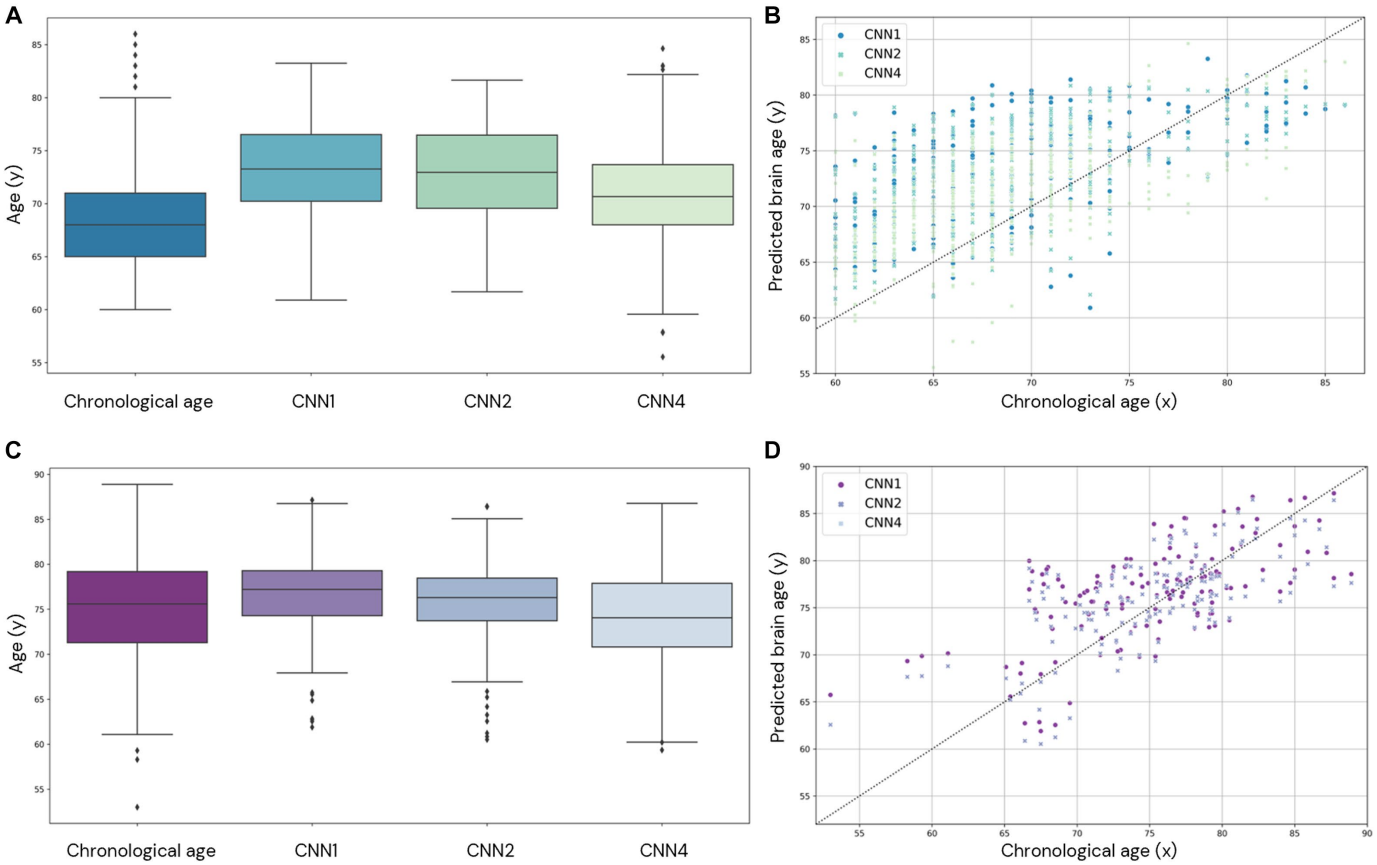
Comparison of brain age prediction distribution for chronological and predicted brain age in CNN1, 2, and 4 for the AddNeuroMed **(A,B)** and J-ADNI **(C,D)** cohorts. In **(A,C)**, we present the boxplot of the predicted brain age for each model compared to the chronological age. In **(B,D)**, the predicted brain age distribution is compared to the chronological age.

**Figure 8:**
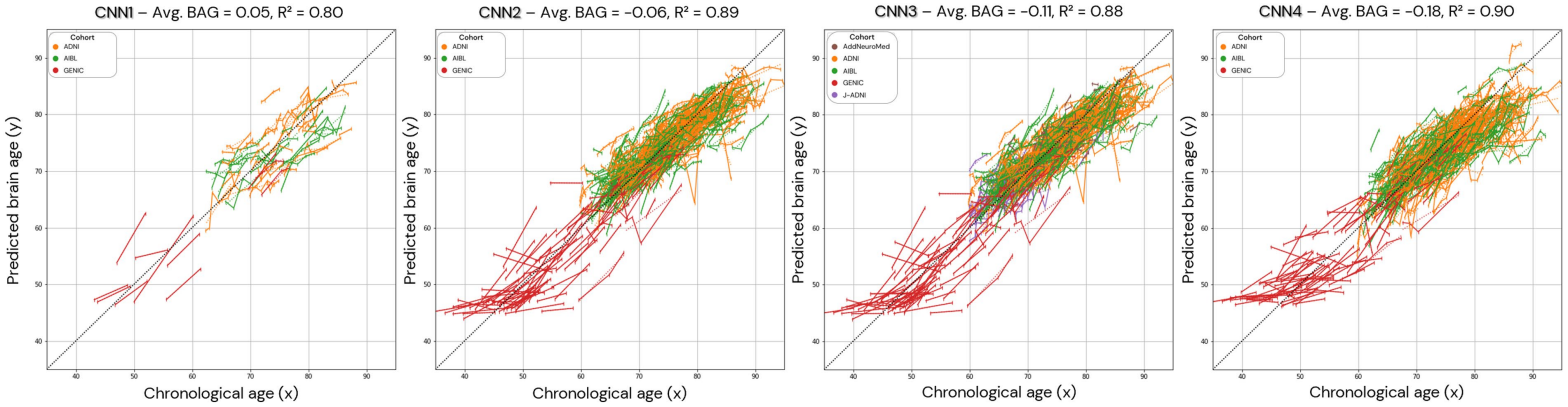
Brain age prediction in longitudinal trajectories for all the models. The average brain age gap/difference (Avg. BAG) in the presented population was calculated and shows a trend towards zero for the brain age difference between predicted and chronological age. In CNN1, only the individuals with longitudinal data in the test set are presented.

Model performance was evaluated through the model’s mean absolute error and correlation coefficients to verify the relationship between chronological and predicted brain age. The results are presented in Figures 4–8, with MAE ranging from 5.63 to 2.25 years and correlation coefficients ranging from 0.77 to 0.90.

Scatterplots of the brain age predictions on the CNN1 (hold-out) approach for the training and testing set of healthy individuals are shown in Figure 4. The results show that there is a strong correlation between chronological and predicted brain age in training (0.83) and test sets (0.80), with MAE = 2.70 years in the training set and MAE = 2.99 years in the testing set.

Scatterplots of the brain age predictions for the CV approach for the CNN2 (CNN1 cohorts), CNN3 (CNN1 cohorts + J-ADNI and AddNeuroMed), and CNN4 (skull-stripped images from CNN1 cohorts) are shown in Figure 5. For CNN2 and CNN3 models, results show a strong correlation (0.83, for both CNNs) between chronological and predicted brain age with a MAE within the lower range of previously published models (MAE = 2.67 years, for both CNNs). However, CNN4 presents a less strong correlation (0.77) and a higher MAE (3.08 years) within the presented models in this study. Age bias-corrected scatterplots of all models are presented in Supplementary material, Section 4, Supplementary Figures S2, S3. Bland–Altman analysis of chronological and predicted brain age is presented in Supplementary material, Section 5. Supplementary Figure S4 illustrates that most of our predictions fit within the two standard deviations, with disagreements between models varying between −0.05 and 0.18.

In order to allow comparison between hold-out and cross-validation approaches, we evaluated only the individuals used in the training/test set of the CNN1 model (hold-out) in the CNN2 (cross-validation approach) (Figure 6). Figure 6A shows the performance of CNN1 (left) and CNN2 (right) using data from the test set. The cross-validation approach (CNN2) shows better performance (smaller MAE) and correlation coefficient (0.86 compared to 0.80 from CNN1) than hold-out. Figures 6B,C illustrate the correlations between brain age predictions for both hold-out (CNN1, *x*-axis) and cross-validation (CNN2, *y*-axis) approaches in the training (left) and test (right) datasets. The correlation of brain age predictions for CNN1 and CNN2 is shown in Figure 6B, while Figure 6C illustrates the correlation between BAG of both models. Both predicted brain age and BAG show a high correlation between models.

We also evaluate the performance of our model in the external datasets AddNeuroMed and J-ADNI (CNN1, 2, and 4). The age prediction distribution is shown in Figure 7 and shows the variability in age prediction for each one of the trained models when applied to unseen data (external dataset).

The calculated MAE of each cohort for all models is presented in Table 1 and ranges from 5.63 to 2.25 years. Normalised MAE and coefficient of determination for each cohort of the trained models can be found in Supplementary Tables S2, S3 and range from 1.04 to 0.35 and 0.89 to 0.55, respectively.

**Table 1:** Calculated MAE for each cohort for each of the trained models.

Model	MAE per cohort (years)					
	ADNI	AIBL	GENIC	UK Biobank	AddNeuroMed	J-ADNI
CNN1^1^	2.56	2.85	4.20	2.75	*4.03*	*5.63*
CNN2^2^	2.40	2.69	3.96	2.66	*3.67*	*5.30*
CNN3^2^	2.41	2.69	3.98	2.67	3.02	2.25
CNN4^1^	2.98	3.33	4.42	3.03	*3.64*	*4.14*

The numbers in italics (AddNeuroMed and J-ADNI cohorts for models CNN1, 2, and 4) were calculated as external datasets for the model. ^1^Statistical differences were found (value of *p* < 0.05) comparing the CNN absolute error; ^2^No statistical differences were found in the comparison between the absolute error of CNN2 and CNN3 (value of *p* = 0.37). Statistical differences between the models were calculated using Friedman’s test with Conover’s post hoc test, adjusting for multiple comparisons with the Bonferroni method.

To understand how our model performed compared to existing models, we further assessed our brain age models’ performance only within the UK Biobank. Then we compared the MAEs we achieved with previous studies that evaluated their models only in the UK Biobank cohort or using different cohorts (Table 2). Our CNN models achieved MAEs ranging between 2.66 and 3.03 years only using images of the UK Biobank cohort. These are very similar to the MAEs achieved by CNN models (Table 2) developed in previous studies (ranging between 2.13 and 4.36). We also present the coefficient of determination between predicted brain age and the identity line for all the available studies.

**Table 2:** Comparison of our models MAE with the literature.

Study	Model	Modality	Preprocessing	MAE	*R* ^2^
Only using the UK Biobank					
Dartora et al.	CNN1	T1	Rigid reg.	2.75	0.88
Dartora et al.	CNN2	T1	Rigid reg.	2.66	0.89
Dartora et al.	CNN3	T1	Rigid reg.	2.67	0.89
Dartora et al.	CNN4	T1	Bias-field and motion cor., Skull-strip., Rigid MNI reg.	3.03	0.85
Bintsi et al. (2020)	CNN (3D ResNet)	T1	Skull-strip., non-linear MNI reg.	2.64	0.77
Bintsi et al. (2020)	CNN (patches)	T1	Skull-strip., non-linear MNI reg.	2.13	0.85
Kolbeinsson et al. (2019)	CNN	T1	Skull-strip., non-linear MNI reg.	2.58	-
Lam et al. (2020)	R-CNN	T1	Bias-field cor., Skull-strip., Rigid MNI reg.	2.86	0.87
Lam et al. (2020)	CNN	T1	Bias-field cor., Skull-strip., Rigid MNI reg.	4.36	0.66
Gupta et al. (2021)	CNN (slices)	T1	Bias-field cor., Skull-strip., Rigid MNI reg.	2.82	-
Dinsdale et al. (2021)	CNN (Female population)	T1	UK Biobank pipeline w/linear registration to MNI	2.86	0.87
Dinsdale et al. (2021)	CNN (Male population)	T1	UK Biobank pipeline w/linear registration to MNI	3.09	0.86
Jonsson et al. (2019)	CNN (transfer learning)	T1	Bias-field cor., Skull-strip., Dartel MNI reg., tissue maps	3.63	0.61
Peng et al. (2021)	SFCN	T1	Bias-field cor., Skull-strip., Rigid MNI reg.	2.14	0.39
Peng et al. (2021)	CNN	T1	Bias-field cor., Skull-strip., Rigid MNI reg.	2.38	-

Study	Model	Modality	Samples/Cohorts	Preprocessing	MAE	*R* ^2^
Using different cohorts						
Dartora et al.	CNN1	T1	16734/4	Rigid reg.	2.99	0.80
Dartora et al.	CNN2	T1	16734/4	Rigid reg.	2.67	0.83
Dartora et al.	CNN3	T1	17296/6	Rigid reg.	2.67	0.83
Dartora et al.	CNN4	T1	16734/4	Bias-field and motion cor., Skull-strip., Rigid MNI reg.	3.08	0.77
Wood et al. (2022)	3D DenseNet	T2	11735/2	Resampling, cropping, padding, intensity normalisation.	3.05	-
Wood et al. (2022)	3D DenseNet	T2	11735/2	Resampling, cropping, padding, intensity normalisation, skull-strip.	3.65	-
Wood et al. (2022)	3D DenseNet	T1	2387/1	Skull-strip., MNI registration	3.83	0.95
Wood et al. (2022)	3D DenseNet	T1	2387/1	“Raw T1”*	4.86	0.90
He et al. (2022)	2D CNN	T1	8379/8	Bias-field cor., field of view norm., Skull-stripp., registration to SRI atlas, cropping.	2.70	-
He et al. (2021)	3D FUS CNN	T1 split	16705/8	Bias-field cor., field of view norm., Skull-stripp., multi-site data harmonisation with histogram matching to SRI atlas, split in two channels (contrast and morphometric), intensity normalisation.	3.00	-
Lee et al. (2022)	3D DenseNet	T1/PET	4127/1	MRI: intensity correction, and segmentation of tissues, intensity normalisation; PET: registration to MRI and MCALT space, scale to SUVRpons, segmentation in meta-ROI.	4.20**	-

CNN, convolutional neural network; CNN1, our CNN model in a hold-out approach; CNN2, our CNN model in a cross-validation approach; CNN3, our CNN model in a cross-validation approach, including two cohorts in the dataset; CNN4, our CNN model in a cross-validation approach, using skull-stripped images; R-CNN, recurrent CNN; SFCN, simple fully convolutional network; *R*^2^ = coefficient of determination. Dartoral et al. refer to the current study. *The authors do not specify if any processing was done. For the “raw” T2 images in the same study, resampling, cropping, and intensity normalisation were performed. **Calculated MAE only for the MRI. SUVRpons, standardised uptake volume ratio in relation to the pons; Meta-ROI, meta region of interest.

To understand the noise levels from our models and their ability to capture subtle changes as a result of the ageing process, we plotted longitudinal trajectories for the participants with multiple time points. The longitudinal predictions, supported by an average longitudinal brain age gap between 0.05 and − 0.18, align with the expected pattern, demonstrating an increase in predicted age over time (Figure 8).

### 3.2 Relevant regions for brain age prediction

**Figure 9:**
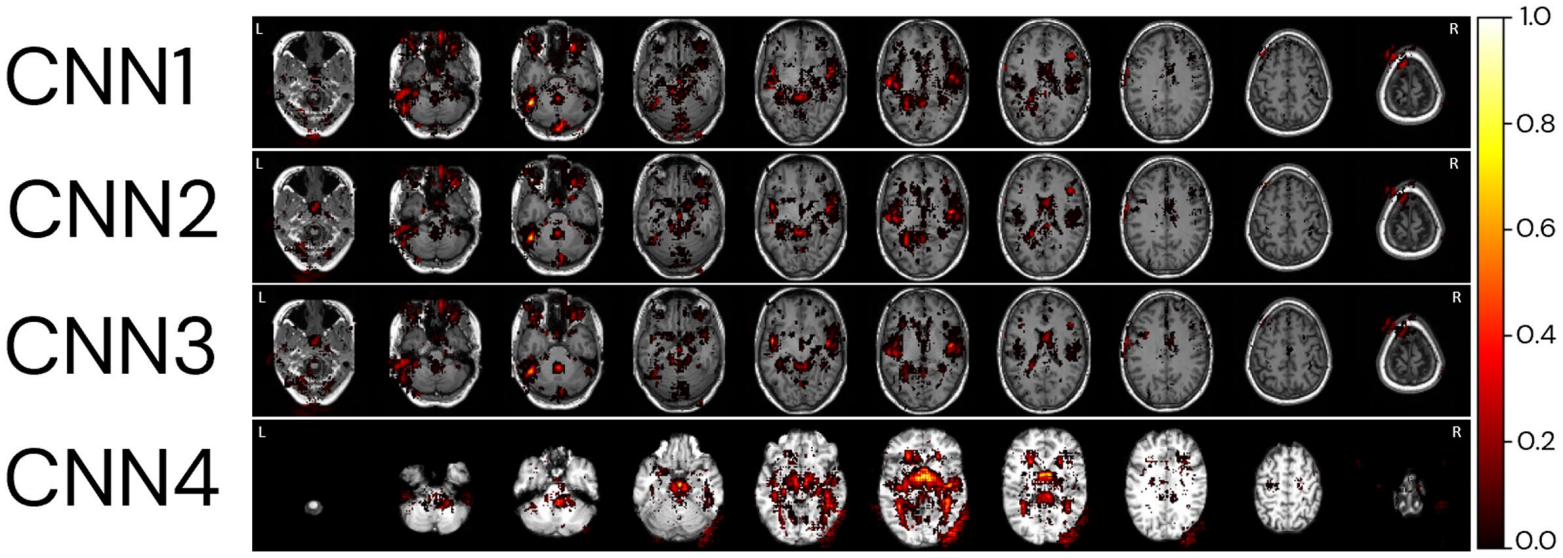
Relevant regions for age prediction. The absolute values of the salience maps for each model were averaged through the whole MRI sample and normalized between 0 and 1 for better visualisation. The SmoothGrad method was used to generate all salience maps. Overlayed salience map colours are normalized for each individual between 0 and 1.

For the explicability of our model, the salience map of each individual prediction was generated. The averaged overlayed salience maps for each CNN model are presented in Figure 9, showing similar patterns of relevant regions for predictions for CNN1-3 and higher variability in these patterns for CNN4. A complementary view of salience map slices is presented in Supplementary Figure S5.

**Figure 10:**
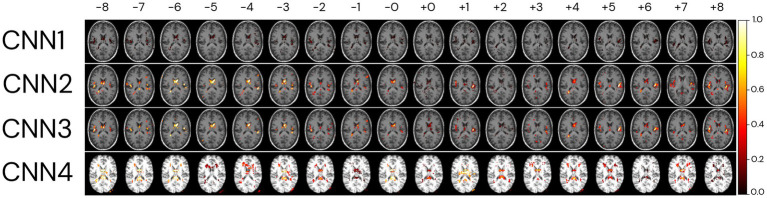
Relevant regions for the brain age prediction based on 1% of the salience maps of individuals with an average age of 65 years overlayed to a random T1w MRI sample for each of the four CNN models. The individuals were randomly selected in the test dataset used in CNN1, according to their brain age difference and proximity to the average age of our sample (65 years). Each row represents the salience maps of one CNN model, where the columns show the same individual’s salience map in each of the four models. The SmoothGrad method was used to generate all salience maps. The brain age difference is shown in the top row of the image, going from −8 to +8 years. Overlaid salience map colours are normalized for each individual between 0 and 1.

To identify regions of importance for predicting biological brain age, 18 individuals were randomly selected according to the following criteria: being in the test dataset of the CNN1, being between 64 and 66 years, and having an age range difference between chronological and predicted brain age in the CNN1 model between −8 and + 8 years (Figure 10). The brain age gap for each individual, predicted in each model, is presented in Supplementary Table S4.

Generally, the averaged salience map across the entire image sample aligns with the highlighted regions in the BAG analysis for the same chronological age (65 years). The BAG analysis reveals similar important regions for predictions, albeit with variations in the intensity of importance amongst them. The negligible (low importance values) importance observed for specific regions may be attributed to the smaller number of images used to train CNN1. It is crucial to note that the BAGs depicted in the top row of Figure 10 are based on CNN1 predictions for the plotted individuals.

In comparison, the CNN4 model exhibits BAG with greater variability than the other three models (CNN1–3). The intensity of importance, with higher importance depicted in yellow colours, proves more significant for predicting younger ages (e.g., −6 years), particularly near the ventricles (Bintsi et al., 2021) and insular cortex (Lee et al., 2022). Conversely, when predicting older ages, greater importance is assigned to the right side of the insular cortex (Lee et al., 2022) and the frontal–occipital region.

### 3.3 Differences in cortical thickness across age groups based on chronological and predicted brain age

**Figure 11:**
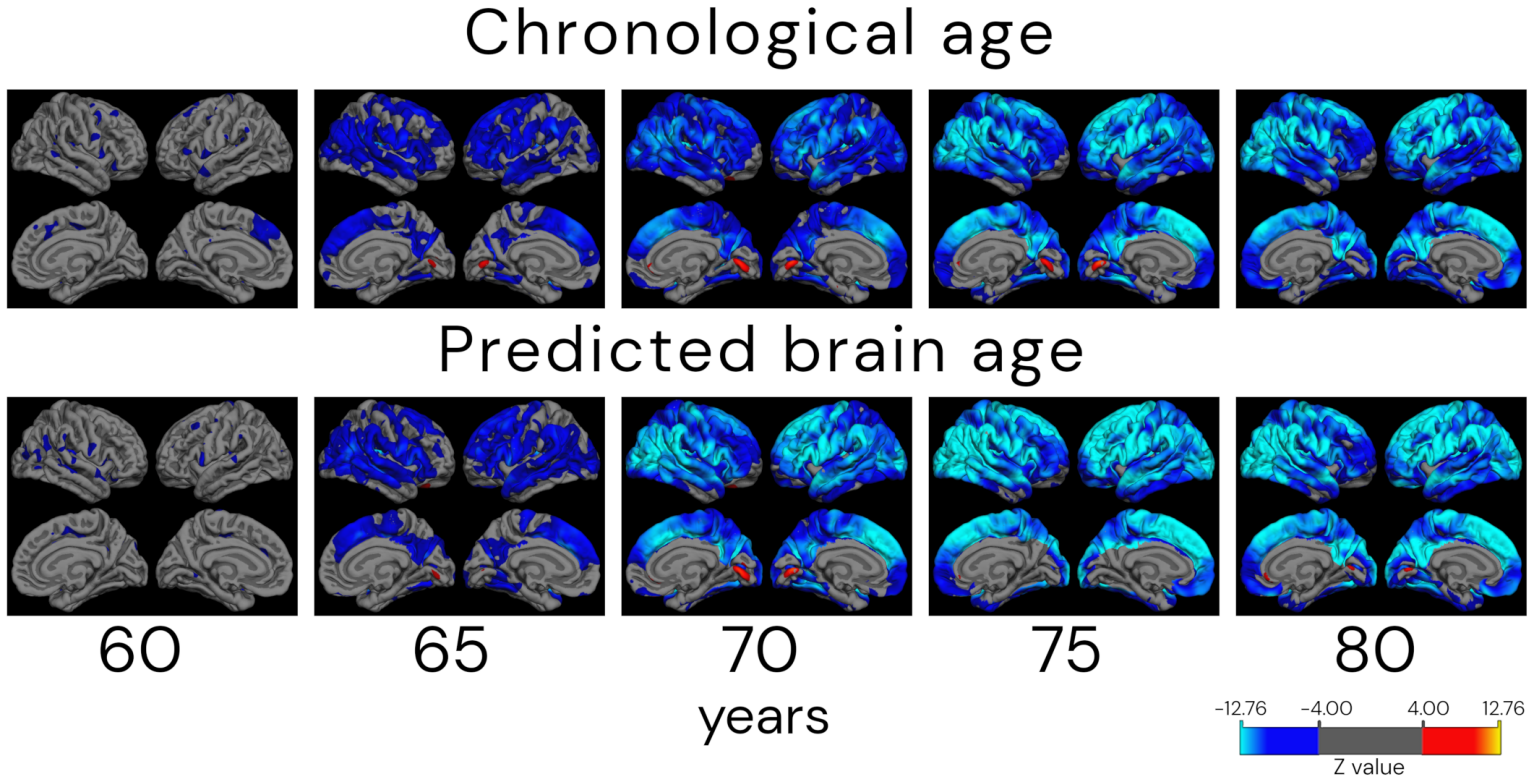
Atrophy patterns in ageing according to chronological and predicted brain age in the cross-validation approach (CNN2). Analysis shows cortical thickness *z*-stat maps corrected for false discovery rate with a threshold of 0.05 of cross-sectional differences between a reference group of 55 (±1.0) years old and older subjects. Individuals were grouped by age ± 1.0 years. The QDEC analyses were done with a 10 mm smoothing kernel and sex added as a covariate. Blue colours represent lower cortical thickness (increased atrophy) in the age group compared to the 55-year-old group, and red colours represent an increased cortical thickness compared to the younger group. It is possible to verify that the atrophy patterns start in parietal–frontal regions in the 65-year-old group. The approach also captures the increased thickness (in red) around the lingual gyrus.

Figure 11 shows the age-related differences in cortical thickness for chronological and biological (predicted) age groups by the CNN2 model. The number of individuals in each age group is presented in Supplementary Table S5. Differences in atrophy patterns between chronological and predicted brain age are observed in the older groups, from the age of 70 years, with mid-frontal and parietal–occipital regions of broader and more widespread differences around the cortex.

## 4 Discussion

In this study, we developed a CNN-based model using one preprocessing step (rigid registration to MNI space), when using new and unseen data in the model, to predict the person’s biological brain age from T1w images to be easily implemented and used. Furthermore, the study was performed as an effort to attend to the highlighted points related to an “ideal” image database (which includes diverse image data) to develop brain age prediction models. Finally, our model evaluation was performed using different approaches—hold-out (CNN1) and cross-validation (CNN2)—and generalisability was tested in external datasets. The usability (CNN3) of the model was also assessed by adding more data (from two external datasets) in the training loop, and to ensure that the previously trained models were using the brain signal for age prediction, we also trained the model using skull-stripped images (CNN4).

Due to the complexity and time-consuming nature of the training networks with large amounts of data, the hold-out method used in CNN1 is the most common approach in the literature (Cole et al., 2017; Jonsson et al., 2019; Lam et al., 2020; Dinsdale et al., 2021; Gupta et al., 2021; Kolbeinsson et al., 2021; Peng et al., 2021; Ren et al., 2022). Our CNN1 model achieved an MAE of 2.99 years in the test set, which agrees with the MAE reported in the literature for hold-out test sets using 1–3 cohorts (MAE of 2.14–4.65 years) but several time-consuming preprocessing steps (Cole et al., 2017; Jonsson et al., 2019; Lam et al., 2020; Dinsdale et al., 2021; Gupta et al., 2021; Kolbeinsson et al., 2021; Peng et al., 2021; Ren et al., 2022). Our calculated MAE is also in the range of the available MAEs of the CNN models (Jonsson et al., 2019; Bintsi et al., 2020; Lam et al., 2020; Dinsdale et al., 2021; Gupta et al., 2021; He et al., 2021, 2022; Kolbeinsson et al., 2021; Peng et al., 2021; Lee et al., 2022; Wood et al., 2022) available in the literature that used hold-out approaches and only data from the UK Biobank but performed several preprocessing steps as opposed to our CNN model. The current debate surrounding the development of brain age models lacks a thorough evaluation of CNN-based brain age models across different external datasets. In such a scenario, it is difficult and relatively unfair to compare the performance of different models without using the same evaluation dataset (Sajedi and Pardakhti, 2019). To overcome such a challenge, we evaluated the performance of our CNN models in two external datasets, AddNeuroMed and J-ADNI, showing that CNN1’s MAE performance is still within a range of 1.5 years when compared to the other CNNs.

Deep learning models, like CNNs, tested in out-of-distribution data result in performance dropping, increasing underestimation in new unseen data. However, including more diverse and variable data in the model’s training increases the model’s robustness/reliability (Mårtensson et al., 2020). One way of including variability in the model is by running it in a cross-validation fashion. We have done this in CNN2, where the same data used for training, validating, and testing CNN1 was used for training a 10-fold cross-validation model (CNN2). In general, the CNN2 model had better performance than CNN1. The correlation with chronological age was higher, the MAE was smaller in CNN2 in all cases of age prediction (training and test sets), and the calculated brain age difference (BAG) was smaller when comparing both models. Comparing the performance of the different cohorts used in the development of this study, we see a tendency for individual smaller MAEs in CNN2. To confirm the robustness of the model, CNN2 was also evaluated in external datasets, confirming the decrease in underestimation, as proved by the increase in the model’s performance in AddNeuroMed and J-ADNI cohorts when compared to CNN1. Even though CNN1 and CNN2 have similar performance in age prediction, the increased variability in the model’s training reduced the average and calculated MAE for each cohort. This is further proved by CNN3, where CNN2 and CNN3 performed similarly (*p* > 0.05). Both models presented the same MAE of 2.67 years and a coefficient of determination of 0.83, but different performances in out-of-distribution data. Specifically, evaluating the predictions in the J-ADNI cohort in CNN3, it is possible to verify that adding the cohort to the training set decreased the predicted MAE for this cohort, going from an MAE of 5.63 in CNN1 to 2.25 in CNN3. This reinforces our hypothesis that the small number of data does not increase the absolute error across all the cohorts (AddNeuroMed and J-ADNI represent ~3% of the total number of images used in CNN3) but increases the performance in a specific cohort. This increases the model’s usability, as data with more variability will be included in the training set, ameliorating the age prediction in external cohorts (Mårtensson et al., 2020). Therefore, additional tests with a greater variety of cohorts are necessary to understand how wide the model’s generalisability is and how it can change by adding more data to the training set.

To ensure that our model was using only brain signals from the T1w MRI and that predictions were not depending on the head morphology or bone tissue, we trained and evaluated CNN4. This model is comparable to CNN2, apart from that skull-stripped brain images were used as input. Regarding performance, CNN2 presented a smaller MAE and a better correlation to chronological age than CNN4. The same tendency is also present in evaluating data only from the UK Biobank. This shows that our CNN works better with minimally preprocessed brain MRIs when compared to heavily preprocessed images. However, for evaluating the external dataset, J-ADNI, CNN4 presented a smaller MAE than CNN2. We hypothesise that using heavily preprocessed images as in CNN4 could remove or decrease the effects of bias field and skull size/format and partially mitigate the inhomogeneity present in the external dataset.

The lower limit of the MAE score is unknown and depends on both the inter-subject variability and age distribution of both training and test datasets. By using stricter exclusion criteria for what is considered “cognitively healthy,” the variability and theoretical MAE lower bound decrease. This makes comparisons between studies of models evaluated on different datasets challenging. Several studies have trained and evaluated their model on the UK Biobank cohort, which enables rough comparisons. However, this restricts the model to cross-sectional image data (at least for the first wave of the UK Biobank data) from a “homogeneous” population from the United Kingdom acquired in standard equipment (Siemens Magnetom Skyra Syngo MR D13) with 3 T MRI following the same protocol, which is not the reality for datasets and clinical/research settings. Also, the performance of CNNs trained on medical images from one cohort may produce systematically different predictions on images outside the training data distribution (Mårtensson et al., 2020). Comparing our results with the literature applied only to UK Biobank images, we observed that using several time-consuming image preprocessing steps, none of the models achieved a MAE smaller than 2.13. The CNN4, which uses skull-stripped images, showed the worst performance within our different approaches using the same CNN architecture. For a more accurate comparison of the model’s performance using the MAE metric, normalised MAE should be used. However, not all the selected papers for comparison presented the average age of the used subgroup of UK Biobank data, limiting the calculation of a normalised MAE. For future comparisons to our study, the normalised MAE for all four different approaches is presented in Supplementary material, Section 6.

Essentially, all our trained models showed MAE levels comparable to those reported in previous literature, which is typically in the range of 2.13–6 (Jonsson et al., 2019; Kolbeinsson et al., 2019; Sajedi and Pardakhti, 2019; Bintsi et al., 2020; Cole, 2020; Lam et al., 2020; Baecker et al., 2021a,b; Dinsdale et al., 2021; Gupta et al., 2021; Tanveer et al., 2022). This indicates that our models have good performance, with the advantage of requiring only one preprocessing step.

A “perfect” model for brain age prediction in cognitively unimpaired individuals should show smooth and non-declining trajectories within the same individual at different time points, assuming that a healthy person’s brain age does not vary rapidly or decrease/increase substantially. Visually, our CNN model in different approaches seems to generate smooth predictions that increase with chronological age, with some noisy predictions deviating from the trajectory. For quantitative analysis, we also calculate the mean age gap (BAG) of all individuals, the mean squared error, and *R*^2^. The models present mean brain age differences between chronological and predicted brain ages smaller than 0.5 years. However, the predictions also show a potential confounder in subsequent analyses of brain age predictions: noise. As it can be observed, for some subjects, fluctuations in brain age differed some years between two scanning sessions that were 1 year apart. It seems unlikely that this is a biological phenomenon, but instead it is attributed to the input data being noisy or of low quality. Most datasets do not have abundant longitudinal data to sort “bad” predictions from “good” predictions. If the data and group sizes are extensive, some level of noise is acceptable and might not affect the interpretation of the results. The maximum average difference between chronological and brain age of all longitudinal plots was −0.11, with *R*^2^’s higher than 0.88. However, this effect can have a non-negligible impact when analysing datasets with small group sizes or running longitudinal analyses with few follow-up scans. This is important to remember when conducting future studies related to brain age, for example, when investigating the association between brain age and neurological disorders with a low prevalence in the population.

The analysis of important brain regions for prediction was realised qualitatively by the plots of the salience maps. The salience maps show that important regions for predicting subjects as older were on the right side of the insular cortex and in the frontal–occipital regions. In contrast, for predicting subjects as younger, highlighted regions are located around the ventricles and insular cortex. The salience maps also show regions symmetrical and asymmetrical, mainly on the left side of the brain (Roe et al., 2021) and around the ventricles (Bintsi et al., 2021), as important for age prediction. Left brain asymmetries with ageing are a typical pattern found in ageing studies of cortical thickness (Koelkebeck et al., 2014; Frangou et al., 2022), cortical volume, and surface area (Koelkebeck et al., 2014). In agreement with Lee et al. (2022), who plotted salience maps for different decades, regions with a higher contribution for age prediction were in the insular cortex (from 30- to 50-year groups), ventricular boundary (50- to 60-year group), and cerebellum (90- to 100-year group). Further studies are necessary to understand why CNN3 has higher variability in the important regions for prediction. They should delve into the nuances of the CNN3 model, which uses the same image data as CNN2 but incorporates additional preprocessing steps.

Interestingly, regions around the eyes were selected in the three non-skull-stripped models (CNN1–3). We hypothesise that changes in the soft tissue and liquid surrounding the orbital space, such as the bony orbit, which has differences in sex (i.e., men usually have a greater skeleton size than women) (Erkoç et al., 2015), as well as general dimensions in orbital structures (Rana et al., 2022), could be used in the model to predict age. For the model using skull-stripped brain images as input (CNN4), right regions close to the cerebellum and occipital lobe, outside the brain, were selected as important for prediction. We believe that the increased noise used for the SmoothGrad in a region that could have a higher neurodegenerative load could be leading to prediction importance outside the brain in this model. More studies are necessary to understand the highlights of this region for prediction. However, we believe this could be an artefact generated by the SmoothGrad method due to the addition of noise in the image for the construction of the maps. Future studies could use different methods to generate salience maps and use different methods to generate skull-stripped images to verify if this outstanding region (right outside the skull region) continues to be highlighted for prediction in this model.

One of the theoretical uses of biological age is for age correction in neuroimaging studies. The hypothesis behind it is that correcting neuroimaging studies for the biological (predicted) age of the individuals will better handle the heterogeneity that we see in ageing, incorporating diversity in longitudinal brain trajectories due to lifestyle, environmental, or even biological factors (Cole et al., 2019; Tian et al., 2023). For the analysis of chronological and biological (predicted) age atrophy pattern differences, we can observe differences mainly in the older groups (as of 70 years). In the comparison of the group of 70 years in chronological and biological (predicted) brain age, atrophied areas of higher statistical significance are present in the mid-frontal to parietal–occipital regions of the group based on biological age. This agrees with the work of Thambisetty et al. (2010), where an anterior–posterior gradient in age-related brain atrophy was found, with frontal–parietal regions showing a greater decline. The groups between 75 and 80 years have more similar atrophy patterns, with higher spread to the parietal lobe in the biological (predicted) brain age. Interestingly, a region of greater thickness in the oldest groups, compared to the reference group (55 years), was found in the primary visual cortex, located in the calcarine sulcus. The shrinkage of the visual cortex is still widely discussed in the literature, with a handful of studies showing cortical thinning of the visual cortex to the sparing of this region. These studies suggest that the visual cortex thickness is use-dependent instead of age-related (Burge et al., 2016; Griffis et al., 2016; Jorge et al., 2020). We hypothesise that this can be a cohort confounding effect, even individuals of 75 years being present in all cohorts, but in larger amounts in ADNI and AIBL. Differences in atrophy patterns between individuals grouped by their chronological and biological (predicted) brain age need to be further studied. However, our results already show different tendencies in atrophy patterns between them. Correcting for biological (predicted) brain age in neuroimaging studies could be one step further in understanding heterogeneity present in ageing and be used in early diagnosis of neurological diseases, prognosis, and even monitoring of treatment response, being one step further to precision medicine.

A key strength of this study is the use of minimally processed images as input for the CNN model, which makes it feasible for implementation in research and, in the long term, clinical settings. Our model requires only registration in MNI space, which typically takes a few seconds and can be easily performed using an open brain image processing software such as FSL or FreeSurfer. Additional preprocessing steps would increase the likelihood of image exclusion during quality control and preprocessing. This would limit the model’s performance and the possibility of using cohorts with a small sample size. CNN models can learn from the image data, including structure and shape, which may not be captured by summary metrics such as volume or segmented tissue maps, without requiring pre-segmented data (Liang et al., 2019; Niu et al., 2020). Our focus on one-step preprocessing, using a rigid registration to the MNI space template previously implemented by Cole et al. (2017), is to ensure accessibility to our model in the future research.

This study includes cohorts to attend to the highlighted points related to, what we believe to be, an ideal image database to develop brain age prediction models. We used detailed information and clinical data for inclusion criteria of many individuals (more than 16,000 T1w MRIs) from different parts of the world (Asia, Australia, Europe, and America) and with a diverse number of image acquisition protocols and MRI scanners covering 1.5 T and 3 T scanners, which increases the model usability due to its generalisation to new unseen data (Mårtensson et al., 2020). This model was compared to the model using only UK Biobank as input with the same acquisition protocol. The results show that the model performance is similar when we include not only different scanners with different magnetic fields but also different acquisition protocols from the different cohorts. We also used all the longitudinal data available and showed that our model presents a reasonable age prediction congruent with the individual timeline. Also, our focus on the cognitive “healthy” status, rather than on the overall health status (e.g., excluding from the training set age-related diseases that affect the body’s organs other than the brain) is a strength of our study as it makes a clear separation between the outcome (brain age) and what leads to that outcome (risk factors, e.g., chronic cardiometabolic disease and risk factors, affective/mood disorders, etc.). Furthermore, to validate the generalisability of the models, we used external datasets of cognitively unimpaired individuals from two cohorts: AddNeuroMed, a cross-European study designed to find biomarkers for Alzheimer’s disease, and J-ADNI, the Japanese version of the ADNI dataset. We chose to use these cohorts because of differences in age distribution, e.g., AddNeuroMed average age is approximately 10 years older than the average of our total sample, different ethnicities, e.g., our sample is composed mainly of European and North American individuals, and both cohorts mostly use images acquired in 1.5 T scanners, whereas the training dataset was based mainly on 3 T MRI, i.e., 96.2% of all images used for training and testing in the CNN1 approach.

Some limitations need to be acknowledged. The large dataset in the current study hindered the possibility of performing extensive quality control. For the CNN model, this would mean inspecting whether the rigid registration was adequate. The random cases we inspected suggested that the overall quality of the segmentations was sufficient. However, tools to automate the quality control process—such as Brusini et al. (2020) and Klapwijk et al. (2019)—will be necessary for future studies on this data size. Regardless of the lack of extensive quality control of the images, our model showed robust findings with only slightly worse performance when compared to previously published works. A potential limitation of this type of model, only trained in cognitively intact individuals, is its performance in neurodegenerative diseases, which warrants further investigation.

Even though we defined the cognitively “healthy” status as consistently as possible across the cohorts, some variation exists, but we acknowledge the clinical and cognitive assessments relied on similar procedures across cohorts. Given our large training set, heavy data augmentation, and running only 20 epochs in training, we minimised the risk of overfitting. However, a crucial consideration for future studies is evaluating how the inclusion of different populations influences brain age prediction models based on minimally processed MRIs. To enhance model robustness, future training models should encompass greater diversity, including cohorts from Asia, Africa, and Latin America, and involve a broader array of scanners (from 1.5 T to 7 T) and imaging protocols (Mårtensson et al., 2020). It is also important to address differences in the distribution of chronological age and the number of subjects in each cohort, as these variations may contribute to overfitting some cohort-specific information and characteristics (e.g., the model may learn that images from GENIC generally fall within the lower age span). Finally, it is worth noting that there might be sociodemographic differences between the cohorts since the recruitment of participants happened in different geographical areas (J-ADNI: Japan, AIBL: Australia, ADNI: North America, and UK Biobank, GENIC, and AddNeuroMed: Europe). However, this is not necessarily a limitation but rather a strength. Indeed, the developed algorithm could be applied in the future research, in which the biological age of the brain is a focus, to some extent independent of the cohort characteristics, thus increasing the generalisability of the model. Future studies need to further test this hypothesis and the impact of different cultural backgrounds on the estimation of brain age.

## 5 Conclusion

In this study, we developed a CNN-based model to predict biological brain age using raw T1w MRI registered to the MNI space, with the goal of accessibility and simplicity in implementation. The model was systematically evaluated using different approaches, comprising several datasets of cognitively healthy individuals with different scans and population characteristics, as well as using cross-sectional and longitudinal data. Our CNN-based model provides results comparable to other validated methods in the literature (a.k.a. state-of-the-art methods) but uses one preprocessing step when using external datasets. The generalisability and usability of the model were tested using external datasets with different demographic characteristics, MRI protocols, and MRI scanners, proving the robustness of the model. In addition, we present the important regions for brain age prediction. We also provide indicators for the use of biological (predicted) brain age as a metric for age correction in neuroimaging studies as an alternative to the traditional chronological age based on the differences in cortical atrophy. Finally, the model’s code and trained CNN weights are made publicly available for the research community to quickly implement and use in their research to study ageing and age-related brain disorders.

## Data availability statement

The original contributions presented in the study are included in the article/Supplementary material, further inquiries can be directed to the corresponding authors.

## Ethics statement

The studies involving humans were approved by Ethics Review Appeals Board DNR 2021-04428. The studies were conducted in accordance with the local legislation and institutional requirements. Written informed consent for participation was not required from the participants or the participants’ legal guardians/next of kin in accordance with the national legislation and institutional requirements.

## Author contributions

CD: Conceptualization, Data curation, Formal analysis, Investigation, Methodology, Software, Validation, Visualization, Writing – original draft, Writing – review & editing. AM: Conceptualization, Data curation, Formal analysis, Investigation, Methodology, Writing – original draft, Writing – review & editing. GM: Conceptualization, Data curation, Methodology, Software, Writing – original draft. GR: Writing – review & editing. JD: Writing – review & editing. J-SM: Data curation, Resources, Writing – review & editing. L-OW: Writing – review & editing. RM: Writing – review & editing. JB: Writing – review & editing. DF: Writing – review & editing. HS: Writing – review & editing. EW: Conceptualization, Funding acquisition, Investigation, Project administration, Resources, Supervision, Writing – review & editing.

## Alzheimer’s Disease Neuroimaging Initiative

Data used in the preparation of this article were obtained from the Alzheimer’s Disease Neuroimaging Initiative (ADNI) database (adni.loni.usc.edu). As such, the investigators within the ADNI contributed to the design and implementation of ADNI and/or provided data but did not participate in the analysis or writing of this report. A complete listing of ADNI investigators can be found at: http://adni.loni.usc.edu/wp-content/uploads/how_to_apply/ADNI_Acknowledgement_List.pdf.

## Australian Imaging Biomarkers and Lifestyle Flagship Study of Ageing

Data used in the preparation of this article was obtained from the Australian Imaging Biomarkers and Lifestyle Flagship Study of Aging (AIBL) funded by the Commonwealth Scientific and Industrial Research Organisation (CSIRO), which was made available at the ADNI database (www.loni.usc.edu/ADNI). The AIBL researchers contributed data but did not participate in the analysis or writing of this report. AIBL researchers are listed at: https://aibl.csiro.au/.

## Japanese Alzheimer’s Disease Neuroimaging Initiative

Data used in the preparation of this article were obtained from the Japanese Alzheimer’s Disease Neuroimaging Initiative (J-ADNI) database deposited in the National Bioscience Database Center Human Database, Japan (Research ID: hum0043.v1, 2016). As such, the investigators within J-ADNI contributed to the design and implementation of J-ADNI and/or provided data but did not participate in the analysis or writing of this report. A complete listing of J-ADNI investigators can be found at: https://humandbs.biosciencedbc.jp/en/hum0043-j-adni-authors.
